# A web-based self-learning system for ultrasound-guided vascular access

**DOI:** 10.1097/MD.0000000000031292

**Published:** 2022-10-28

**Authors:** Daisuke Sugiki, Hisao Matsushima, Takayuki Asao, Joho Tokumine, Alan Kawarai Lefor, Toshirou Kamisasanuki, Mitsuhiro Suzuki, Sayaka Gomei

**Affiliations:** a Emergency and Critical Care Center, Dokkyo Medical University Saitama Medical Center, Saitama, Japan; b Gunma University Center for Mathematics and Data Science, Gunma, Japan; c Department of Anesthesiology, Kyorin University School of Medicine, Tokyo, Japan; d Department of Surgery, Jichi Medical University, Tochigi, Japan.

**Keywords:** central venous catheter, peripherally inserted central catheter, self-learning, simulation education, ultrasound-guided, vascular access, web-learning

## Abstract

Ultrasound-guided vascular access is practiced widely. Optimal educational methods have not yet been established. We hypothesized that a step-by-step web-based learning system is effective for self-learning. In this study, we examined the potential of this system as a self-learning tool. This was an observational study at a single institution. Participants included residents, who were self-educated through the web-based system. Skill proficiency was measured after self-learning. The primary outcome was the extent to which self-learning enabled residents to acquire proficiency in the basic skills of ultrasound-guided vascular access: needle visualization, hand-eye coordination, and avoiding posterior wall penetration. A secondary outcome was the time required to achieve proficiency. Thirty-nine residents were enrolled in this study. Eleven residents (28%) passed the first skill assessment test. There was no significant difference in the number of days that the web-based system was accessed, the total number of screen views, or the total learning time between participants who passed and those who failed the first test. Skill assessment scores between those who passed and those who failed the first test were different, especially the score for hand-eye coordination, and the number of posterior wall penetrations. Self-learning with a web-based system enabled 28% of residents to pass the first skill assessment test. The remaining 72% failed the first skill assessment test but continued to learn using the web-based system and eventually passed the test. Hence, the web-based system needed formative testing to function as a self-learning system. Simulation education for vascular access is expected to increase in educational content and methods. Self-learning through a web-based learning system is a leading candidate for this growth.

## 1. Introduction

Ultrasound-guided central venous catheterization (US-CVC) has become the “gold standard” technique for central venous catheterization and is mandated in many institutions.^[1–4]^ US-CVC has been shown to improve the success rate and reduce the complication rate for this procedure.^[5]^ However, appropriate training is required to derive the benefits of US-CVC,^[6,7]^ and a consensus has been reached on minimum training requirements.^[8]^ Schmidt et al showed the importance of needle visualization and hand-eye coordination as required skills to perform successful catheterization.^[9]^ We previously validated the importance of avoiding posterior vein wall penetration for preventing mechanical complications of central venous catheterization.^[10]^ Hence, acquiring skills in 3 areas, needle visualization, hand-eye coordination, and avoiding posterior wall penetration may be essential for safe central venipuncture in clinical practice.

We developed a minimum-skill requirement integrated teaching system to acquire these skills.^[11]^ This teaching system was constructed in a step-by-step manner, resulting in improved needle tip visualization and puncture accuracy, with a higher success rate. Currently, this system is usable via the Internet.^[12]^ Recently, self-learning using the Internet has been tried and its usefulness reported.^[13]^ We hypothesized that using a web-based learning system proceeding in a step-by-step manner would be beneficial for self-learning. In this study, we examined the utility of this educational system as a self-learning tool.

## 2. Methods

This study was approved by the local ethical committee (the Clinical Research Institutional Review Board of Dokkyo Medical University Saitama Medical Center, approval number 1883). Participants (from May 2019 to July 2020) were recruited from among first-year residents by advertising a self-learning program for US-CVC. This was designed as a single-group observational study. Written informed consent was obtained from all participants. Exclusion criteria included prior experience in ultrasound-guided vascular access, and refusal to participate.

The primary outcome was whether someone naive for central venous catheterization can acquire the 3 necessary skills (needle visualization, hand-eye coordination, and avoiding posterior wall penetration) through self-learning using the web-based learning system, or not. A secondary outcome was the amount of time needed to achieve the skills. Another secondary outcome was to determine which of the required skills was most difficult to learn.

### 2.1. Self-learning using the web-based learning system

This web-based self-learning system provides on-line learning content for the short-axis out-of-plane and the long-axis in-plane techniques for US-CVC. The content includes 463 chapters, including explanations and 80 minutes of instructional video.

**Figure 1.**
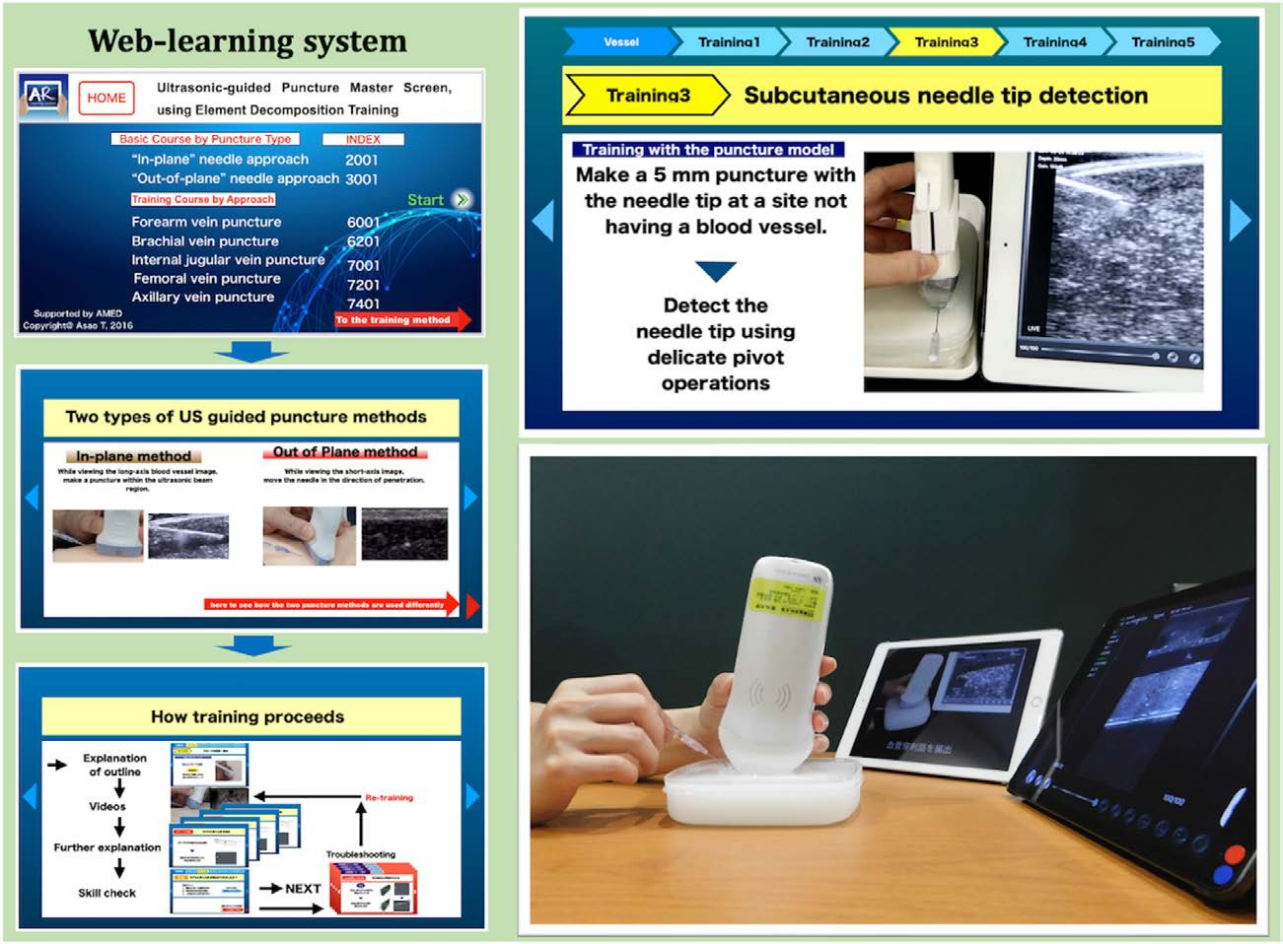
Web-based learning system for ultrasound-guided vascular access. Access to the web-based learning system allows step-by-step simulation training such as skill explanation, training videos, skill tips, and evaluation tasks. All web-page pictures are reproduced from Ref. 12 with the permission of the copyright holder. US = ultrasound.

The needle used in this study was a cannula-over-the-needle type (size; 20G, length; 32 mm, Surflo I.V. Catheter, Terumo Co., Tokyo, Japan), which is not a needle for clinical placement of central venous catheters, in consideration of cost and the technique for placing an indwelling peripherally inserted central catheter. Ultrasound imaging with a 10MHz linear probe was used (Isono, Alfabio Co., Gunnma, Japan). A tablet computer (iPad, Apple Japan Inc., Tokyo, Japan) was used as an ultrasound monitor. The simulator consists of a box-shaped container filled with agar gel and a simulated vessel (inner diameter: 6 mm, depth: 5 mm, AGL800UGP-GEL, Alfabio Co., Gunnma, Japan). Access to the web-based learning system was obtained using a tablet computer (Fig. 1). Information to access the web site^[12]^ and the evaluation scoring system for the exercise (JAMS CVC-Instructor’s guide ver. 5, Summarized in Supplemental file 1 -3, http://links.lww.com/MD/H695: http://links.lww.com/MD/H696: http://links.lww.com/MD/H697)^[14]^ were provided to each participant at least 1 week prior to the actual skill assessment test.

### 2.2. Skill assessment test and data collection

Needle tip visualization was evaluated based on visibility of the needle tip and shaft just before puncturing the simulated vessel. Hand-eye coordination was assessed to ensure that the needle tip could always be delineated by coordinating the movement of the probe with the ultrasound image. Puncture of the posterior wall of the simulated vessel was evaluated using a puncturing skill evaluation device (Puncture Accuracy Measuring Device, Alfabio Co., Gunnma, Japan) which uses an endoscope inserted into the simulated vessel to confirm the presence of the needle in the simulated vessel.^[11]^ The criteria for passing the test are 3 points or more for needle visualization (Supplemental file 1, http://links.lww.com/MD/H695 & 2, http://links.lww.com/MD/H696, left panel) and hand-eye coordination (Supplemental file 1, http://links.lww.com/MD/H695 & 2, http://links.lww.com/MD/H696, right panel), and the absence of posterior wall penetration (Supplemental file 3, http://links.lww.com/MD/H697). Skill assessment tests were performed for both the short-axis out-of-plane (Supplemental file 1, http://links.lww.com/MD/H695) and long axis in-plane (Supplemental file 2, http://links.lww.com/MD/H696) techniques. Assessment was performed by an expert in US-CVC (HM).

Technical advice for each skill was given to each participant after skill assessment. If the resident could not pass the skill assessment test, re-test was scheduled for another day. Skill assessment scores for each test were recorded for each resident. The individual score sheet for each resident was checked to evaluate passing the skill assessment test. All records on the score sheet were transferred to a computer as anonymous data and analyzed later. The score sheets were subsequently shredded. This web-based learning system provides learning logs, which record individual user’s learning time, access date and time, and the content viewed. The screen views are organized like a slideshow, so users proceed through the slides one by one. Each slide contains photos, explanations, videos, and other information. Time spent accessing and using the web-based learning system were recorded and analyzed later.

### 2.3. Statistical analysis

Statistical analyses were performed using the SPSS statistical package (version 28.0.0.0(190), IBM Corp, Armonk, NY). Mann–Whitney’s *U* test was used to evaluate the total learning time, the total number of screen views, and the number of days that the system was accessed before assessment. A *P* value <.05 was considered statistically significant.

## 3. Results

Forty participants enrolled in this study. One participant enrolled in the self-learning system, but did not complete the assessment, resulting in data from 39 participants being analyzed in this study. No participant had previous experience with US-CVC.

**Figure 2.**
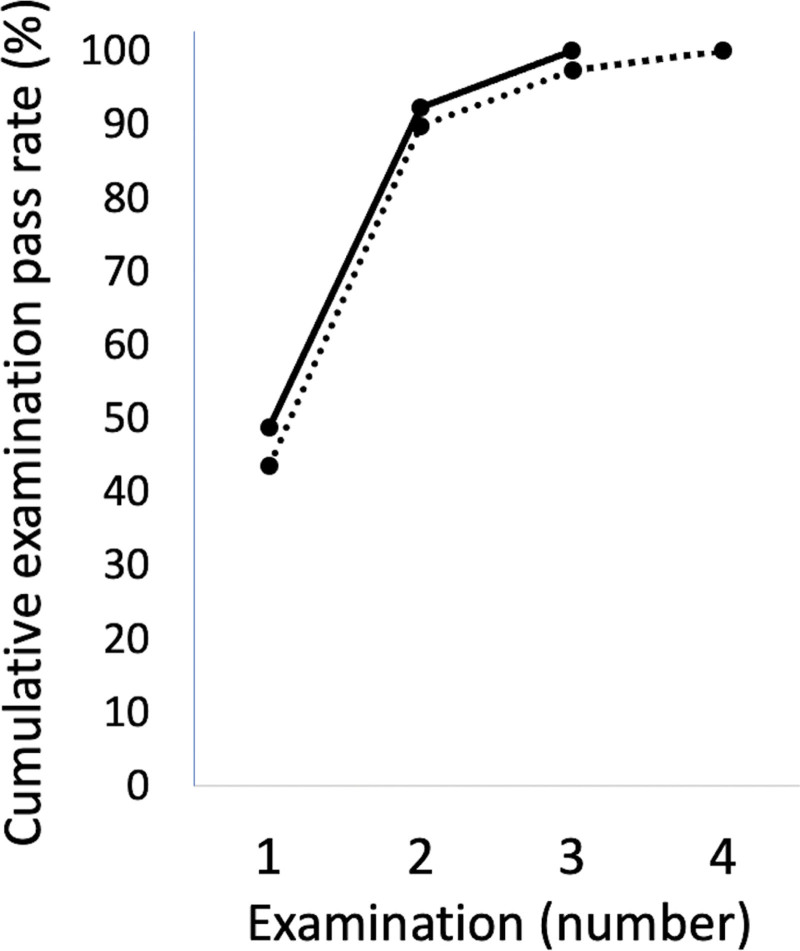
Cumulative examination pass rate. The cumulative examination pass rate following additional examinations. Solid line: in-plane approach, Dashed line: out-of-plane approach.

Eleven participants (11/39, 28%) passed the first skill assessment test, 21 participants passed the second test (21/39, 54%), 6 participants passed the third test (6/39, 15%), and 1 passed the fourth test (1/39, 3%). Finally, all participants passed the skill assessment test (Fig. 2).

There was no significant difference in the number of days that the web-learning system was accessed, the total number of screen views, or the total learning time among those participants who passed the first assessment test and those who failed (Table 1). The skill assessment scores for those who passed the first assessment test and those who failed were different, especially the score for hand-eye coordination, and the presence of posterior wall penetration (Table 2).

**Table 1:** Total learning time, number of screen views, and number of days accessed before the skill assessment test.

	Pass	Fail	*P* value
Participants	11	28	
Total learning time (min, mean ± SD)	71 ± 51	77 ± 95	.45
Total number of screen views (min, mean ± SD)	205 ± 136	204 ± 206	.75
Number of d accessed (d, mean ± SD)	2.3 ± 1.8	2.6 ± 2.8	.89

SD = standard deviation.

**Table 2:** Skill assessment scores.

Skill assessment score	Technique	Passed	Fail
Needle visualization median (IQR)	In-plane	4 (3–4)	3 (2–4)
	Out-of-plane	4 (3–4)	3 (2–3)
Hand-eye coordination median (IQR)	In-plane	4 (4–4)	2 (2–3)
	Out-of-plane	4 (3–4)	2 (2–2)
Posterior wall penetration number (%)	In-plane	0 (0%)	5 (18%)
	Out-of-plane	0 (0%)	6 (21%)

IQR = interquartile range.

## 4. Discussion

A web-based self-learning system enabled 28% of participants to pass the skill assessment test. The remaining 72% failed the skill assessment test but continued to learn through the web-based system and eventually passed the test. Hence, the web-based self-learning system can be used as a tool to acquire the 3 skills necessary for ultrasound-guided vascular access. However, the results also show that self-learning cannot be completed only using a web-based learning system. Objective assessment by an expert was needed to determine whether skills were acquired or not. The results of this study do suggest that effective training is possible with a web-based learning system and strongly supports the value of a web-based learning system as an educational tool.

The web-based learning system has been used in our institution as a teaching aid for mastery learning and has the characteristics needed for mastery learning. These include clearly defined learning objectives, subject content arranged in difficulty levels, essential learning items, and continuity of learning.^[15]^ However, the web-based learning system needed formative testing to function as a mastery learning system. The skill assessment test was conducted to examine whether a web-based learning test could be used for self-learning, but as a result, the skill assessment test functions as a formative assessment.

Schmidt et al identified 2 required skills to perform successful catheterization, which include needle visualization and hand-eye coordination.^[9]^ The present study shows that hand-eye coordination seemed to be more difficult to acquire than needle visualization. Comparing both skills, needle visualization is a static skill, while hand-eye coordination is a dynamic skill. Hand-eye coordination requires needle visualization in motion. Therefore, hand-eye coordination is a more advanced skill than needle visualization. This contributes to the difference in skill observed between those who passed the first test and those who did not.

When US-CVC was not widely used, Blaivas et al reported that the short axis out-of-plane approach was easier to master.^[16]^ However, Blaivas et al also warns against misidentification of the needle tip and shaft in the out-of-plane approach.^[17]^ Stone et al reported that needle tip visibility with the in-plane approach was better than with the out-of-plane approach.^[18]^ Tokumine et al reported on the mechanism of lateral venous wall penetration using the long axis in-plane approach.^[19]^ The results in the present study also showed no difference in the rate of posterior wall penetration between the in-plane and out-of-plane approaches. This can be explained by the previous report by Tokumine et al^[19]^ which showed that accidental penetration of the lateral wall close to the posterior wall can occur even with the in-plane approach. These results suggest that the risk of posterior wall penetration is present in both approaches, which may be why there is no clear superiority with either approach.^[20]^ In conclusion, understanding the characteristics of the 2 approaches will help set the ultimate educational goal for proficiency.

Posterior wall penetration occurs when the needle is moved. Posterior wall penetration can occur with needle visualization techniques alone. For this reason, Schmidt et al proposed that hand-eye coordination techniques are necessary,^[9]^ and the guidelines followed this proposal.^[8]^ In evaluating these 2 techniques in the present study, the absence of posterior wall penetration was considered a part of the final result.

The results of this study are encouraging but require further evaluation. The generalizability of the results of this study is limited by the applicability of skills obtained with simulation education to clinical practice. In particular, the basic skill set to be achieved in this study, namely needle visualization, hand-eye coordination, and avoiding posterior wall penetration, can be considered to be a minimum requirement and at a level that allows for application in ideal clinical settings. Expert supervision of novice operators is needed in clinical practice. Studies of learning curves have begun to consider proficiency in the clinical practice of US-CVC.^[21,22]^ Further evaluation of this web-based self-learning system with other groups of participants at varied levels of training is needed.

Okano et al reported in a meta-analysis that simulation education for vascular access was of questionable value to improve clinical practice.^[23]^ This may be due to a lack of training in techniques to specifically avoid ultrasound pitfalls, which often occur while moving the interlocked probe and needle. The occurrence of these events also stems from the physical characteristics of a vein. The walls of a vein are very compliant, and when the internal pressure of the vein is low, tenting occurs, causing the needle to simultaneously press against the anterior and posterior walls of the vein and eventually penetrate. In a report of closed claims in Japan, perforation of the posterior wall of the vein often results in accidental puncture of the artery behind the vein, and reflux due to bleeding may be observed. In clinical settings, residents are taught that confirmation of blood backflow is the most important evidence of a successful puncture, but it is also a sign that should not be relied upon alone. In the present study, a training program was devised to improve basic ultrasound-guided techniques. Improved ultrasound manipulation techniques are expected to increase the success rate of puncture and at the same time to prevent complications. In the present study, backflow of blood after puncture was intentionally not considered so that users would have to depend on other evidence.

This web-based system was created to provide logical learning content for ultrasound-guided vascular access. The results of the present study suggest that objective evaluation is necessary for skill acquisition when this system is used alone for self-learning. In recent years, technological innovation in image analysis has been progressing. If artificial intelligence is able to evaluate ultrasound guiding techniques in the near future, it will be possible to incorporate this functionality into the software of a web-based educational system to enable skill acquisition through self-learning alone.

## 5. Conclusions

This web-based learning system in its present form is incomplete for self-learning. However, this system was effective when used for hands-on training seminars for ultrasound-guided vascular access.^[11]^ To use this system for self-learning, further studies are needed to incorporate objective assessment of skills into the system.

The need for simulation education for vascular access is clear,^[24,25]^ but its effectiveness remains unclear.^[23]^ Simulation education for vascular access is expected to continue to grow in educational content and method.^[26]^ Self-learning through a web-based learning system is one of the leading candidates.

## Author contributions

Conceptualization: Daisuke Sugiki, Hisao Matsushima.

Data curation: Daisuke Sugiki, Toshirou Kamisasanuki, Mitsuhiro Suzuki, Sayaka Gomei.

Formal analysis: Daisuke Sugiki.

Funding acquisition: Takayuki Asao.

Investigation: Daisuke Sugiki, Hisao Matsushima, Toshirou Kamisasanuki, Mitsuhiro Suzuki, Sayaka Gomei.

Methodology: Daisuke Sugiki, Hisao Matsushima.

Project administration: Hisao Matsushima, Takayuki Asao.

Supervision: Hisao Matsushima.

Writing – original draft: Daisuke Sugiki, Joho Tokumine.

Writing – review & editing: Alan Kawarai Lefor.

## Supplementary Material


