# Supplementary material for: A web-based self-learning system for ultrasound-guided vascular access
Source: Medicine (Baltimore). 2022 Oct 28;101(43):e31292. doi: 10.1097/MD.0000000000031292 (PMC9622633; doi:10.1097/MD.0000000000031292)
Supplement: Supplementary file 1 [file medi-101-e31292-s001.pdf]

Supplemental file 1: Skill Assessment Score [14] ***Out-of-plane approach***

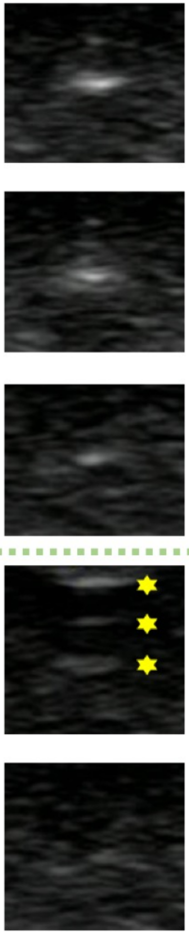

5: *Clearly visible*

4: *Visible*

3: *Blurred*

2: *Indistinguishable*

1: *Invisible*

Needle visualization

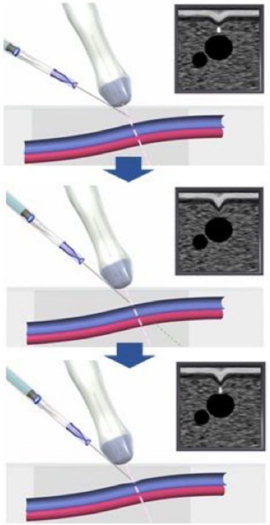

5 : *Excellent real-time handling*

4: *Good almost real-time*

3: *Fair clumsy*

If the needle tip is advanced beyond the ultrasound beam, determine Score 2.

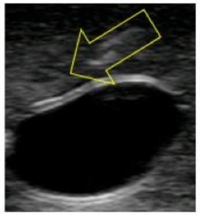

2: *Poor wrong angle*

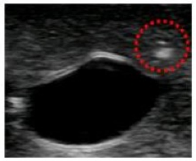

1: *Unacceptable wrong direction*

Hand-eye coordination
