# Supplementary material for: A web-based self-learning system for ultrasound-guided vascular access
Source: Medicine (Baltimore). 2022 Oct 28;101(43):e31292. doi: 10.1097/MD.0000000000031292 (PMC9622633; doi:10.1097/MD.0000000000031292)
Supplement: Supplementary file 2 [file medi-101-e31292-s002.pdf]

Supplemental file 2: Skill Assessment Score [14] ***In-plane approach***

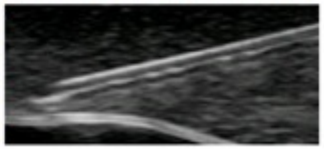

5: Clearly visible

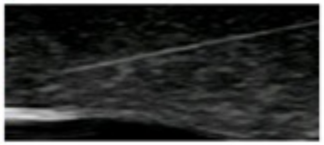

4: Visible

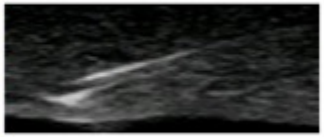

3: Needle tip visible

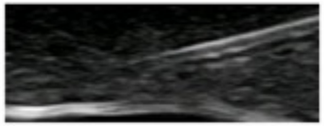

2: partially visible

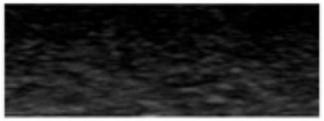

1: Invisible

Needle visualization

5 : Excellent
always visible
needle visualization 5 or 4

4: Good
visible
needle visualization 4 or 3

3: Fair
interrupted visible
needle visualization
3 at penetrating the anterior vein wall

2: Poor
interrupted visible
needle visualization
2 at penetrating the anterior vein wall

1: Unacceptable
invisible

Hand-eye coordination
