# Supplementary material for: A web-based self-learning system for ultrasound-guided vascular access
Source: Medicine (Baltimore). 2022 Oct 28;101(43):e31292. doi: 10.1097/MD.0000000000031292 (PMC9622633; doi:10.1097/MD.0000000000031292)
Supplement: Supplementary file 3 [file medi-101-e31292-s003.pdf]

Supplemental file 3: Skill Assessment Score [10] ***No posterior wall penetration***

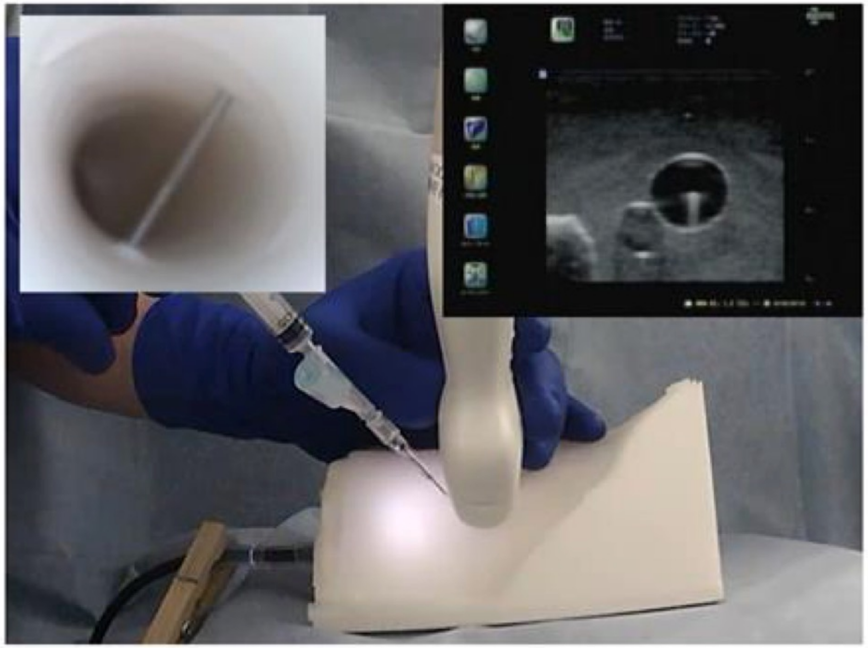

posterior wall penetration

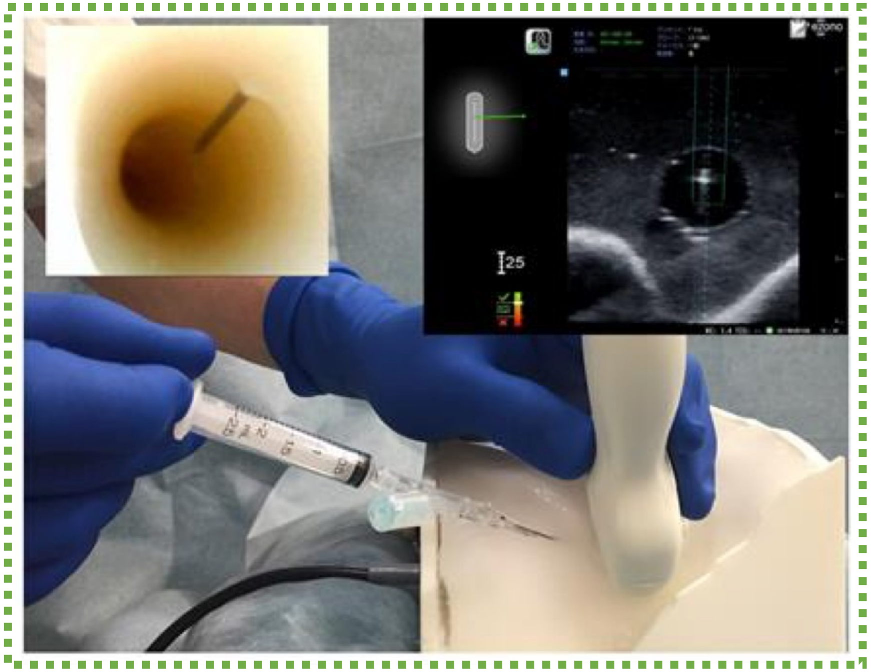

No posterior wall penetration
